# CLASV: Rapid Lassa virus lineage assignment with random forest

**DOI:** 10.1371/journal.pntd.0013512

**Published:** 2025-09-09

**Authors:** Richard Olumide Daodu, Ebenezer Awotoro, Jens-Uwe Ulrich, Denise Kühnert

**Affiliations:** 1 Center for Artificial Intelligence in Public Health Research, Robert Koch Institute, Wildau, Germany; 2 Department of Mathematics and Computer Science, Freie Universität Berlin, Berlin, Germany; Public Health Agency of Canada, CANADA

## Abstract

Lassa fever, caused by the Lassa virus (LASV), is a deadly disease characterized by hemorrhages. Annually, it affects approximately 300,000 people in West Africa and causes about 5,000 deaths. It currently has no approved vaccine and is categorized as a top-priority disease. Apart from its endemicity to West Africa, there have been exported cases in almost all continents, including several European countries. Distinct Lassa virus lineages circulate in specific regions, and have been reported to show varying immunological behaviors and may contribute to differing disease outcomes. It is therefore important to rapidly identify which lineage caused an outbreak or an exported case. We present CLASV, a machine learning-based lineage assignment tool built using a Random Forest classifier. CLASV processes raw nucleotide sequences and assigns them to the dominant circulating lineages (II, III, and IV/V) rapidly and accurately. CLASV is implemented in Python for ease of integration into existing workflows and is freely available for public use.

## Author summary

Lassa fever is a viral illness that causes yearly outbreaks in West Africa and has the potential to spread internationally, with cases recently reported in several countries outside the region. Quickly identifying the genetic lineage of the virus responsible is critical for inferring the source of outbreaks and guiding medical and public health responses. However, tools designed specifically for rapid Lassa virus lineage identification are almost non-existent. In this study, we introduce CLASV, a fast and user-friendly tool that uses machine learning to identify the major circulating lineages of Lassa virus from genetic sequences. Validated on real-world data, CLASV achieved high accuracy even when working with incomplete sequences. It runs in minutes on a standard computer, making it practical for deployment in local low-resource laboratories. By providing timely and reliable lineage information, CLASV has the potential to enhance outbreak response and enable more targeted public health interventions—both in West Africa and in countries managing imported cases.

## Introduction

Since its discovery in 1969, Lassa fever (LF) has constituted a major public health threat, with 100,000–300,000 cases and around 5,000 deaths annually in West Africa [1]. Although the overall fatality rate is relatively low (~1%) [1], fatality is reportedly high among pregnant women and fetuses [2–4]. Experimental research involving LASV is confined to Biosafety Level 4 (BSL-4) laboratories, and the disease is currently on the World Health Organization’s (WHO) top priority list [5]. Although transmission is typically confined to West Africa, exported LASV cases have been reported worldwide [6], including Europe and Asia, emphasizing the need for robust biosecurity measures, rapid diagnosis, effective treatments, and vaccines. The global threat remains persistent, as demonstrated by the most recent confirmed case reported in China [7].

There are currently seven known lineages, each with a largely distinct geographic range. Lineages I–III circulate in separate regions of Nigeria [8], lineage IV is found in Sierra Leone, Guinea, and Liberia [9], lineage V in Mali and Côte d’Ivoire [10], and lineage VII in Togo and Benin [11,12]. A strain discovered in *Hylomyscus pamfi* in Nigeria was designated lineage VI [13]. These lineages differ in immunogenicity [14–16] and may contribute to variations in disease severity, as suggested by observed geographic disparities [9] and supported by animal model data [17]. Host genetics factors, such as mutations in glycosyltransferase-encoding gene *LARGE1*, which have been associated with milder disease in Nigerian patients [18], may also influence clinical outcomes.

With the decreasing cost of next-generation sequencing and the World Health Organization actively supporting the expansion of genomic surveillance capacity across Africa [19], real-time pathogen sequencing is poised to become integral to outbreak response. Yet, bioinformatic resources tailored to African pathogens remain scarce. At present, lineage assignment during outbreaks typically relies on traditional phylogenetic methods [7,9,20]. While these approaches are essential for long-term evolutionary analysis, they require expert annotation and in-depth knowledge of LASV lineages, making them impractical in emergency situations.

Fast lineage assignments can be performed using various methods, including alignment-based approaches [21,22] or k-mer-based sequence classifiers [23,24]. These approaches typically classify new sequences by mapping them against lineage-specific reference genomes. However, such methods often face challenges, including defining consistent classification thresholds, selecting appropriate reference sequences, and maintaining robustness at lower taxonomic levels. A more sophisticated option is adapting the Nextclade online platform for rapid LASV lineage assignment [25,26]. This approach proved effective during the SARS-CoV-2 pandemic – used alongside machine learning (ML)–based tools such as pangoLEARN and the phylogenetic placement engine pUShER [27,28]. Although phylogenetic placement methods generally outperform ML classifiers in accuracy, ML‐driven techniques remain far superior in speed [28]. Crucially, ML methods can learn directly from data, eliminating the need for manually defined classification rules or cutoffs, which can be difficult to design and maintain for rapidly evolving pathogens. Moreover, standardized evaluation metrics in ML classification foster reproducibility and trust. For LASV, it is especially important to provide a portable tool that can operate offline in field settings where data are sensitive or internet access is limited. Beyond Lassa virus, rapid lineage assignment is so important that, despite the SARS-CoV-2 pandemic being declared over, studies about rapid SARS-CoV-2 lineage assignment are still forthcoming [29].

Here we introduce CLASV, a novel, fast, and user-friendly lineage-assignment pipeline that enables (i) rapid inference of an outbreak’s geographic origin, (ii) early indication of potential lineage-specific clinical risk, and (iii) standardized data for molecular epidemiology. CLASV uses a Random Forest classifier trained on curated Lassa virus glycoprotein precursor (GPC) sequences - a part of the virus that plays a major role in cell entry and immune response [30,31]. It provides accurate lineage assignments within minutes on a standard computer.

The rationale for developing CLASV stems from the urgent need for accessible, portable, and reproducible Lassa virus lineage-typing tools that can be deployed in both endemic and non-endemic regions. By addressing critical public health gaps in Lassa fever surveillance, CLASV has the potential to empower frontline laboratories and international reference centers to rapidly integrate lineage data into clinical decision-making, preparedness for imported cases, and targeted public health interventions. Such capability could significantly enhance outbreak preparedness, optimize patient management, and strengthen overall responses to Lassa fever outbreaks.

Github: https://github.com/JoiRichi/CLASV, PIP installation: https://pypi.org/project/CLASV/

## Methods

### Data and preprocessing

LASV sequences released up until December 1, 2023, along with their accompanying metadata, were downloaded from NCBI Virus (available at https://www.ncbi.nlm.nih.gov/labs/virus/vssi/#/). To ensure the inclusion of only field samples, the “exclude lab strain” filter was applied. Using the GPC gene from the reference ID NC_004296, we extracted and aligned the GPC regions from all sequences using LAST [32] and MAFFT [33](available at https://mafft.cbrc.jp/alignment/server/specificregion-last.html), resulting in 1,021 GPC sequences. Sequences with more than 5% gaps and ambiguous nucleotides of the total alignment length were removed, reducing the dataset to 808 sequences. The final stop codon position was removed from the alignment because the stop codon signals the termination of translation and does not encode an amino acid. Sequences lacking sampling dates and locations were excluded, leaving 753 sequences.

Alignment visualization and exploration were conducted using Aliview [34]. Manual curation was performed to ensure codon consistency. Specifically, misalignment involving codons disrupted by three gaps were adjusted by moving a single nucleotide to match the two others to ensure proper translation. Nucleotide to amino acid translation was performed using Aliview. All data analyses were conducted in Python using standard libraries such as pandas [35](available at https://pandas.pydata.org/), scikit-learn [36], and Biopython [37]. The workflow was implemented using Jupyter Notebook in Google Colab [38].

### Sequence Annotation

**Fig 1:**
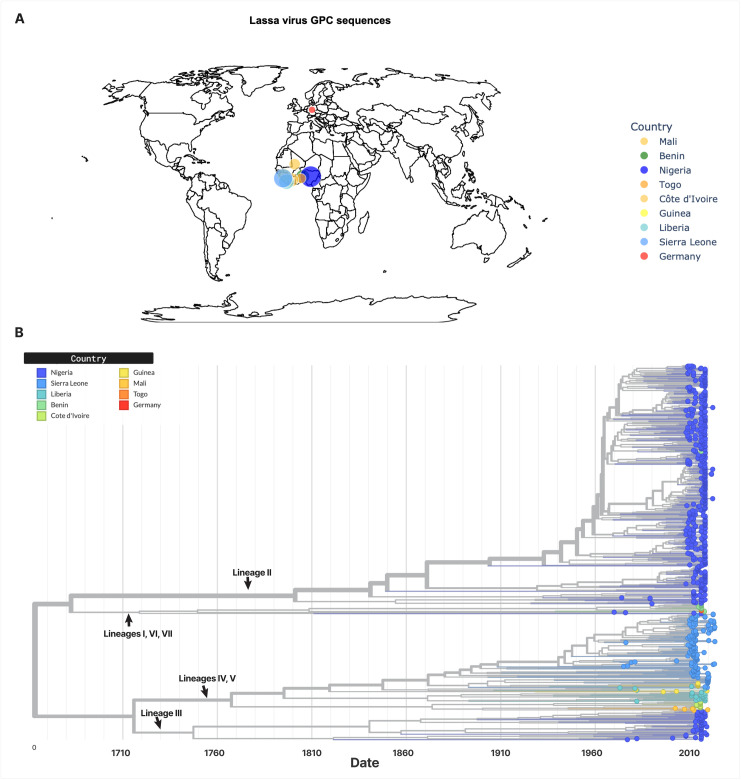
Lassa virus geographic distribution and phylogeny. **(A)** Geographic distribution of Lassa virus glycoprotein (GPC) sequences used in this study. Circle size reflects the number of submissions per sampling location, with counts log-normalized for visualization purposes. Basemap data were obtained from Natural Earth (public domain; https://www.naturalearthdata.com/downloads/50m-cultural-vectors/50m-admin-0-countries-2/.; terms: https://www.naturalearthdata.com/about/terms-of-use/; accessed 22 August 2025). The map was generated using Plotly (https://plotly.com/python/map-configuration/). (B) Phylogenetic tree of nucleotide sequences annotated by lineage (black arrows). Leaf nodes are coloured by geographical location. Clipart from OpenClipart (https://openclipart.org/detail/239915/mouse-cursor-pointer).

We extracted sampling locations from the accompanying GenBank metadata. From the 753 sequences, 542 sequences originated from Nigeria, 141 from Sierra Leone, 11 from Guinea, 1 from Germany, 3 from Togo, 24 from Liberia, 5 from Mali, 13 from Côte d’Ivoire, and 13 from Benin. To assign lineages, we reconstructed a phylogenetic tree (Fig 1) and annotated its clades based on the literature [8–13] (see Supplementary GitHub data). We labeled the leaves of each clade, based on the annotations of their corresponding sequences and classified them into the following lineages: 480 sequences for lineage II, 59 sequences for lineage III, 194 sequences for lineages IV and V combined, and 16 sequences for lineage VII. Lineages I and VI were excluded due to insufficient data. Lineages IV and V were grouped together based on the recommendation of Whitmer et al. [11], who noted that the distance between these two lineages is similar to those between other sublineages. During model preparation, we discovered that some sequences, despite having distinct GenBank IDs and metadata, were identical. To avoid data leakage, we deduplicated the dataset, resulting in a total of 618 sequences for model training—comprising 387 sequences of lineage II, 50 of lineage III, 169 of lineages IV and V combined, and 12 of lineage VII.

### Phylogenetic reconstruction of the LASV GPC

Using a pipeline based on Snakemake [39], Nextstrain [26], and Augur [40], we reconstructed both a Maximum Likelihood tree and a time tree. The alignment and metadata, which include sampling dates, country, and host information, served as inputs to the pipeline. A Maximum Likelihood tree was constructed using IQ-TREE [41] through the application programming interface (API) provided by Augur. Similarly, using the Augur API, the tree was processed by TreeTime [42], together with the metadata, to generate a time-calibrated tree.

The tree and accompanying metadata were parsed using the Augur export command into a JSON file, which was subsequently visualized using Auspice (available at https://auspice.us/), part of the Nextstrain toolkit. Phylogenetic images were generated using Auspice and then edited alongside all figures for clarity using Figma (available at https://www.figma.com/).

### Sequence encoding

**Fig 2:**
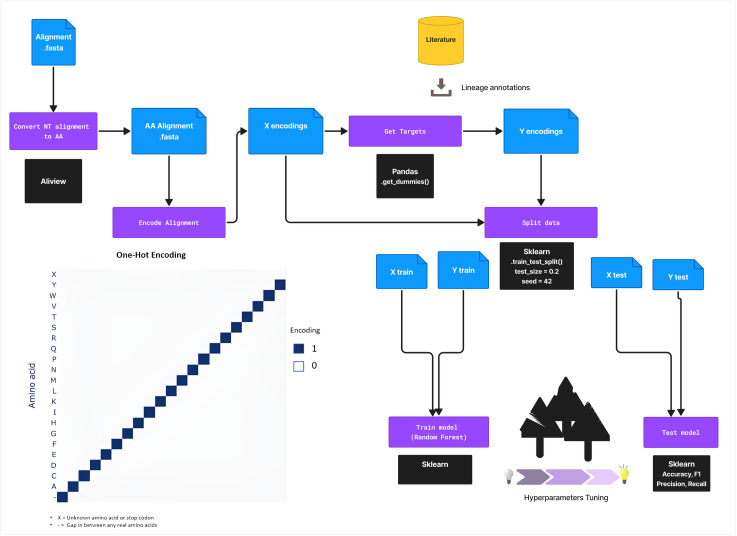
Training workflow. Multiple DNA sequence alignment of the glycoprotein encoding gene sequences is converted into an amino acid alignment. The resulting amino acid sequences were one-hot-encoded and labeled with the assigned lineages. Then, the data was split into a training and test data set for training and evaluating the Random Forest Classifier. To achieve optimal performance from the model, hyperparameter tuning was conducted. Clipart from OpenClipart (https://openclipart.org/detail/220988/light-bulb-on-off; https://openclipart.org/detail/305300/download).

We started with the amino acid alignment, comprising 618 sequences and 491 positions. Gaps preceding and following real amino acids were converted to ‘unknowns’, as these typically reflect short sequences or sequencing errors and thus lack biological meaning. To prevent any bias, all features and targets were one-hot encoded. More precisely, each amino acid is encoded by a vector of length 21, representing the 20 possible amino acids and an alignment gap. Here, a vector containing only zeros encodes for an ‘unknown’ amino acid (Fig 2). We subsequently flattened the encoded features, with each position in the vector transformed into an individual column, resulting in a data matrix of dimensions 618 by 10,311. Finally, we labeled each sequence with its corresponding lineage, using a one-hot-encoding for the lineages.

### Model training

We selected a Random Forest (RF) model for lineage classification, implemented using the scikit-learn package. RF was chosen for its effectiveness in managing class imbalance [43].

Hyperparameters were optimized using a grid search strategy with 5-fold stratified cross-validation. During parameter tuning, we evaluated combinations of model depth (using maximum depths of 10, 20, and unlimited depth), number of trees (100, 500, and 1000 estimators), and the minimum number of samples required to split a node (2, 5, 10, and 20). Each configuration was assessed using mean cross-validation (CV) accuracy as the scoring metric. The random state for the RF model was set to 80 to ensure reproducibility. Based on the grid search results, the final lineage classification model used 100 decision trees, a minimum split size of 2 samples, and no restriction on maximum depth.

### Model evaluation

Following hyperparameter tuning, we selected and evaluated the best-performing model configuration using stratified 5-fold cross-validation. For each fold, macro-averaged precision, recall, and F1-score were calculated alongside overall accuracy. After cross-validation, we retrained the final model on the complete training dataset and then assessed its performance on a stratified 20 percent holdout test set (Fig 2). All metrics, including accuracy, precision, recall, F1 score, balanced accuracy, and the Matthews correlation coefficient, were computed using the scikit-learn package [36].

## Results

**Fig 3:**
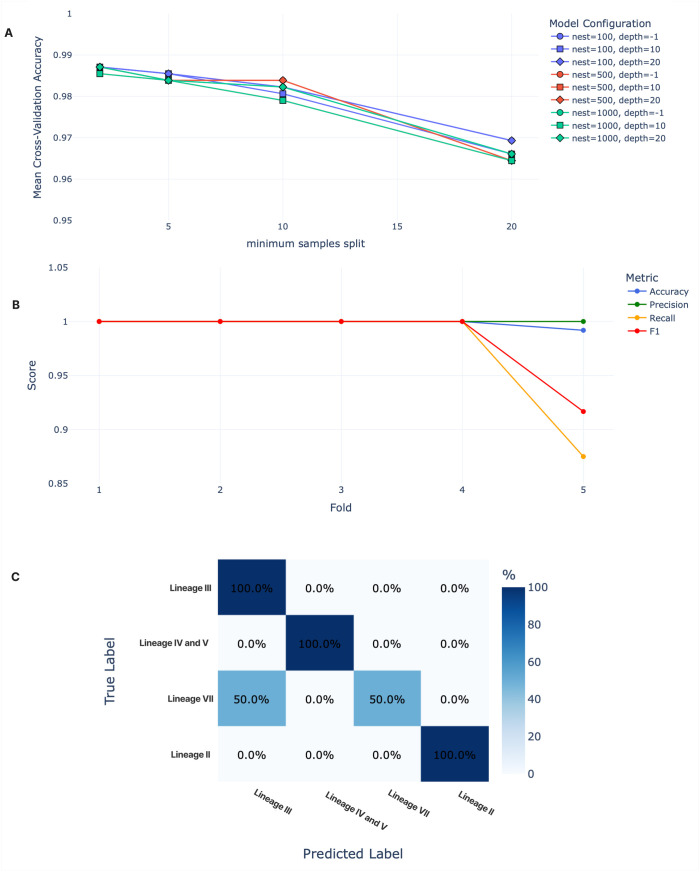
Model performances. **(A)** Grid search for the best Random Forest configuration. A forest of 100 trees with unlimited depth (represented as –1 in the graph) achieved the highest mean CV accuracy, while increasing the minimum sample split consistently reduced performance. **(B)** Five-fold stratified cross-validation using these best parameters shows 100% accuracy in folds 1–4, with a drop only in fold 5. **(C)** Investigation of fold 5 reveals that the model underperforms specifically in classifying lineage **VII.**

We developed a Random Forest classification pipeline, CLASV, which reliably predicts the major LASV lineages II, III, IV/V (Fig 3). The overall accuracy of the underlying model is approximately 0.99. Predictions for lineage VII are less reliable, likely due to small sample size. When validated on independent real-life datasets containing lineages II and IV/V, CLASV achieved excellent performance, correctly classifying all sequences in both sets (Fig 5).

**Fig 4:**
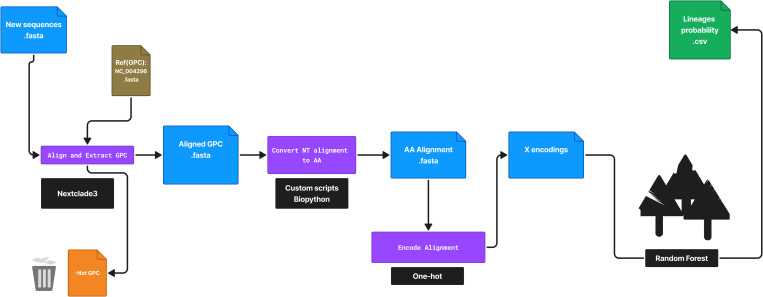
Classification workflow. CLASV first extracts the GPC region from the input sequences using Nextclade3 (see Methods). The aligned nucleotide sequences are converted into amino acid sequences and encoded similarly to the model’s training process. Subsequently, the encoded sequences are run through the trained random forest model, which predicts the probability per lineage. The highest probability is taken as the final prediction, provided it is greater than 0.5. Sequences with highest probability below or equal to 0.5 are classified as inconclusive.

**Fig 5:**
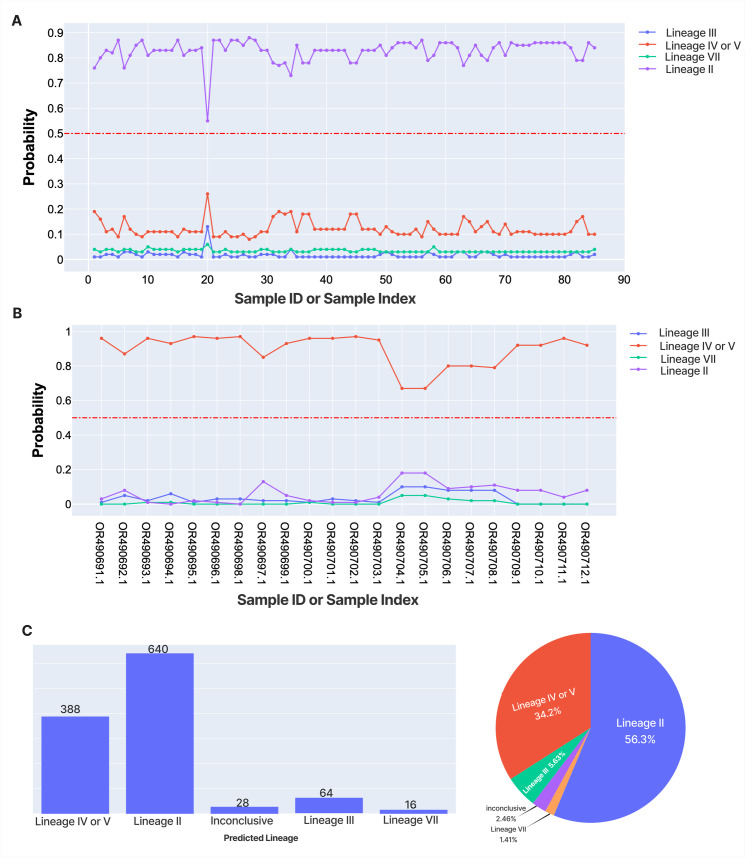
Performance of CLASV. **(A)** The pipeline accurately and precisely predicts sequences provided by Adesina et al. as belonging to Lineage II, despite the sequences being around half the size of the GPC. **(B)** CLASV accurately and precisely predicts sequences provided by Bangura et al. as belonging to Lineage IV or V. **(C)** The predicted distribution of LASV lineages in GenBank as of 06/07/2024. The inconclusive predictions include KM822128 (Pinneo), which belongs to lineage I. This reinforces the belief that lineage I is divergent from other lineages and emphasizes the pipeline’s ability to avoid false positives (supplementary GitHub code). Over half of all LASV GPC sequence in Genbank is predicted to belong to Lineage II. These figures are auto-generated for every run, enabling rapid evaluation of the resulting lineage classification.

### Hyperparameter Optimization and Model Selection

We performed a 5-fold stratified grid search to optimize three primary Random Forest hyperparameters: maximum tree depth (set to 10, 20, or unlimited), the number of estimators (100, 500, or 1,000), and the minimum number of samples required to split an internal node (2, 5, 10, or 20). Across all tested combinations, model performance consistently declined as the minimum sample split increased (Fig 3A). The highest mean cross-validation accuracy, approximately 98.7%, was achieved by several configurations. However, based on the best_params_ attribute of the GridSearchCV class [36], which we used in evaluating accuracy and precision in parallel, the top-ranked configuration in both scenarios consisted of 100 estimators, unrestricted tree depth, and a minimum number of samples required to split a node of 2.

These results indicate that fine-grained decision boundaries are critical for accurately classifying LASV lineages based on GPC sequence features. Allowing trees to grow without depth restriction likely enabled the model to represent subtle, lineage-specific variation embedded within the high-dimensional one-hot encoded sequence data. The superior performance observed at the lowest minimum split threshold suggests that rare but informative sequence motifs contribute meaningfully to classification and are more readily captured when node splitting is unconstrained. In contrast, higher minimum split thresholds likely limited the model’s ability to partition the feature space around these sparse signals, introducing bias and reducing overall accuracy.

### Cross-validation performance

The selected model, configured with the best-performing hyperparameters, demonstrated consistent performance across all five CV folds (Fig 3B). Macro-averaged precision was uniformly 1.0 across folds, while accuracy, recall, and F1-score showed a slight decrease in fold 5 (Fig 3B).

### Class-specific performance

Analysis of the confusion matrix for fold 5 (Fig 3C) revealed perfect classification for Lineages II, III, and the combined group of Lineages IV and V. However, half of the Lineage VII samples were misclassified as Lineage III, reducing recall for that class and thereby lowering the macro-averaged F1-score. Since lineage VII is by far the least represented in the dataset, our model seems to lack enough information to learn the most important features to correctly classify this lineage.

### Deployment and further validation of CLASV

The final Random Forest model was trained using 100 estimators, with all other hyperparameters retained at their default values (scikit-learn version 1.6.1; see Fig 2). The test set, which comprised 20 percent of each lineage and was processed identically to the training data, yielded perfect classification for lineages II, III, and the combined IV and V group: accuracy, precision, recall, and F1 score each reached 100 percent for these lineages. However, likely due to the very small lineage VII sample size (12 in total), the model underperformed on lineage VII, achieving a recall of 0.50 and an F1 score of 0.67. Overall accuracy on the entire test set was 99.19 percent, balanced accuracy was 0.875, and the Matthews correlation coefficient was 0.9847. The final model was packaged into a Python pipeline called CLASV (Fig 4).

To further validate CLASV, we applied the model to more recently published datasets from Adesina et al. [44] and Bangura et al. [45], which included 85 and 22 GPC sequences, respectively. None of these sequences were used during training or testing. Notably, the Adesina et al. dataset included incomplete GPC sequences, with the shortest being 663 nucleotides—less than half of the expected ~1,473 nucleotide full GPC length. Despite this, CLASV accurately classified all sequences from both studies with 100% precision and accuracy (Fig 5).

Finally, to evaluate runtime performance, we processed the complete publicly available LASV GPC dataset. On a standard machine (Darwin 24.4.0, Apple M1 Pro, 16GB RAM) using 1 core, CLASV completed classification in less than 30 seconds. Results are summarized in Fig 5, and full implementation details are available in the accompanying GitHub repository.

## Discussion

As Lassa fever continues to cause annual outbreaks in West Africa and exported cases are increasingly reported globally, the ability to determine viral lineages quickly is critical for both clinical management and public health response. Although the World Health Organization supported a major expansion of genomic sequencing capacity across the continent during the COVID-19 pandemic [19], there remains a significant gap in accessible, locally relevant bioinformatic tools for African pathogens [46,47].

In this study, we address this need by introducing CLASV, a rapid and reliable tool for LASV lineage assignment. CLASV is implemented in Python and uses the Snakemake workflow [39]. It accepts raw sequence input and performs classification using a Random Forest model trained on curated amino acid alignment. The pipeline delivers results for up to a thousand sequences in under two minutes on a standard computer (Fig 5), making it practical for both local laboratory deployment and integration into larger surveillance platforms. Its modularity supports seamless incorporation into broader genomic workflows, bolstering ongoing bioinformatics capacity-building efforts in resource-limited settings [48,49].

Model performance was consistent and robust, achieving perfect classification (100% accuracy, precision, recall, and F1-score) on held-out test data in lineages II, III, IV/V, and fully accurate assignments on external datasets not used during training (Fig 5A, 5B). This high level of performance reflects the careful curation of training alignment and the distinct sequence characteristics of the major LASV lineages. The tool performed equally well on complete and partial GPC sequences (Fig 5A), suggesting resilience to real-world variability in sequence quality. However, CLASV can only classify Lassa virus sequences that contain the GPC, and thus, cannot detect recombination and reassortment events.

Given the recurrent nature of LASV outbreaks [29], rapid and reliable lineage classification is a critical component of timely outbreak detection and response. However, the need for rapid lineage assignment is not limited to LASV. Other high-consequence zoonotic viruses endemic to Africa—such as Ebola, Marburg, and Crimean-Congo hemorrhagic fever—would benefit from the development of similar pathogen-specific tools. While sequencing capacity has improved substantially [19], parallel investments in accessible software and workforce training remain essential [47,50]. Our workflow can be adapted readily to classify any pathogen for which curated sequence alignment and categorical annotations are available.

Because no dedicated LASV lineage annotation tool is currently available in the NCBI submission pipeline, CLASV could be embedded as a pre-submission hook, automatically annotating incoming LASV sequences and returning standardized lineage tags before GenBank release.

The available genomic LASV data across lineages is highly imbalanced (Fig 5C). A current limitation resulting from this imbalance is that the pipeline supports only four lineages: II, III, IV/V (combined), and VII. The model is also not reliable in classifying lineage VII, as shown in Fig 3. This is most likely due to the dataset containing too few sequences of lineage VII (n = 12). Most misclassifications were to lineage III, although phylogenetic evidence suggests that lineage VII GPC is closer to lineages I and VI, which were excluded from our analysis [11] (Fig 1). Consequently, the classifier will require periodic retraining as more sequences become available and as additional lineages are formally defined. The workflow is designed to accommodate such updates easily, and adoption of standardized LASV lineage naming criteria [11] would further support reproducibility and cross-study compatibility.

Finally, we emphasize the need for expanded sequencing efforts and standardized lineage assignment protocols. These actions will not only improve our understanding of LASV evolution but also support the development and long-term utility of tools like CLASV in both research and applied public health contexts.
